# Next-Generation Sequencing Approaches in Genome-Wide Discovery of Single Nucleotide Polymorphism Markers Associated with Pungency and Disease Resistance in Pepper

**DOI:** 10.1155/2018/5646213

**Published:** 2018-01-09

**Authors:** Abinaya Manivannan, Jin-Hee Kim, Eun-Young Yang, Yul-Kyun Ahn, Eun-Su Lee, Sena Choi, Do-Sun Kim

**Affiliations:** ^1^Vegetable Research Division, National Institute of Horticultural and Herbal Science, Rural Development Administration, Jeonju 55365, Republic of Korea; ^2^Department of Vegetable Crops, Korea National College of Agriculture and Fisheries, Jeonju 54874, Republic of Korea

## Abstract

Pepper is an economically important horticultural plant that has been widely used for its pungency and spicy taste in worldwide cuisines. Therefore, the domestication of pepper has been carried out since antiquity. Owing to meet the growing demand for pepper with high quality, organoleptic property, nutraceutical contents, and disease tolerance, genomics assisted breeding techniques can be incorporated to develop novel pepper varieties with desired traits. The application of next-generation sequencing (NGS) approaches has reformed the plant breeding technology especially in the area of molecular marker assisted breeding. The availability of genomic information aids in the deeper understanding of several molecular mechanisms behind the vital physiological processes. In addition, the NGS methods facilitate the genome-wide discovery of DNA based markers linked to key genes involved in important biological phenomenon. Among the molecular markers, single nucleotide polymorphism (SNP) indulges various benefits in comparison with other existing DNA based markers. The present review concentrates on the impact of NGS approaches in the discovery of useful SNP markers associated with pungency and disease resistance in pepper. The information provided in the current endeavor can be utilized for the betterment of pepper breeding in future.

## 1. Introduction

Pepper is a diploid and self-pollinating plant belonging to the genus* Capsicum* in the Solanaceae family. In general, the Solanaceae family comprises more than 3,000 different species which include plants with notable economic importance such as potato, tomato, tobacco, and petunia. Most of the plants in Solanaceae consist of similar chromosome number (*n* = 12) with different genome sizes [1]. Pepper or chili has been first domesticated by Native Americans around 6000 BC [2]. Till date, the spicy nature of the pepper is widely used in global cuisines. Among the pepper varieties,* Capsicum annum*,* Capsicum baccatum*,* Capsicum chinense*,* Capsicum frutescens*, and* Capsicum pubescens* are predominantly cultivated [3]. The consumption of pepper is growing exponentially due to its organoleptic and nutraceutical properties. Pepper consists of vital medicinal properties such as antioxidant [4], anticancer [5], anti-inflammatory [6], and antiobesity properties [7]. Owing to its increasing demand, the cultivation of pepper has been increased by 40% in the last decade. Although the pepper cultivation is increasing, the disease affecting the production of* Capsicum* is also rising alarmingly. In particular, the incidence of bacterial and fungal infections causes drastic yield loss in pepper. Therefore, in order to address the above-mentioned problems, various breeding technologies incorporating novel approaches utilizing the next-generation sequencing technologies have been considered as the promising method. The primary improvement in the molecular marker assisted breeding or genomics-assisted breeding of pepper has become more reliable after the availability of the* Capsicum* genome sequence information.

An enormous increase in the understanding of trait specific characteristics associated with various molecular markers can be well-studied with the genomic information. In recent days, the genome-wide molecular markers have been identified in pepper. Among the DNA based molecular markers, single nucleotide polymorphisms (SNPs) markers revolutionized the molecular breeding in various crops [8]. Due to their high density occurrence in the plant genomes, the utilization of SNPs has gained a major importance in the molecular marker assisted breeding [8]. Moreover, the availability of several computational methods and genotyping approaches to efficiently detect and validate SNPs in the genome aids in the identification of vital SNPs associated with trait of interest. In plant genome, the discovery of SNP is a complicated process due to the presence of repetitive elements [9]. However, recent advancements in the NGS technologies enhanced the high throughput discovery of SNPs. The transcriptome resequencing acts as an inexpensive and rapid method for the identification of SNPs even in the complex plant genomes such as avocado, canola, eucalyptus, maize, and sugarcane [10–14]. Moreover, the authentication of discovered SNPs using various genotyping platforms guarantees the utilization of the predicted SNP marker for the identification of polymorphism among the species. Several high throughput SNP genotyping techniques such as Golden Gate assay based on Illumina's BeadArray technology, Infinium assays, TaqMan approach in association with OpenArray platform, competitive allele specific polymerase chain reaction, high-resolution melt assays, and fluidigm based SNP validation are available for accurate SNP genotyping [15–18]. Moreover, the SNP markers aid in the understanding of genome-wide fine mapping of genes and linkage between a trait and marker and enables high-resolution quantitative trait loci (QTL) mapping [8]. Recently, Hulse-Kemp et al. [19] have established a haplotype map of the* C. annuum* genome using the SNP Infinium array technology. The report suggested the importance of the next-generation resequencing approaches in cohort with the SNP markers for the development of novel tools for the pepper breeding. In detail, the report discusses the discovery of various SNP markers using resequencing approach to create PepperSNP16K array and validated it in several mapping populations. According to Hulse-Kemp et al. [19], the PepperSNP16K array can be utilized for the rapid assessment of numerous markers and QTLs for the improvement of valuable traits in pepper. Likewise, Taranto et al. [20] analyzed the population structure and genetic diversity in* Capsicum* using the SNP markers discovered using genotyping by sequencing approach. The GBS based discovery of SNP markers in a collection of* Capsicum* subspecies resulted in the identification of genetic diversity in 222 cultivated pepper genotypes [20]. Hence, the GBS methodology has provided a reliable tool for the discovery of high-quality SNPs for the investigation of population structure in pepper germplasm collection [20]. Moreover, the study has also applied clustering algorithms such as Bayesian and Hierarchical methods for the efficient understanding of the population structure in pepper genotypes [20]. In addition, the study has also implied the advantages of SNP markers in the development of modern plant breeding in pepper with economically important horticultural traits. Thus, it can be highly appreciated that the synergistic application of SNP markers along with the modern genomics approaches and NGS technologies accelerates the improvements in the molecular-genomics-assisted plant breeding. Hence, the upcoming sections deal with the discovery of SNP markers associated with important traits in pepper.

## 2. Pepper Genome: A Brief View

**Figure 1:**
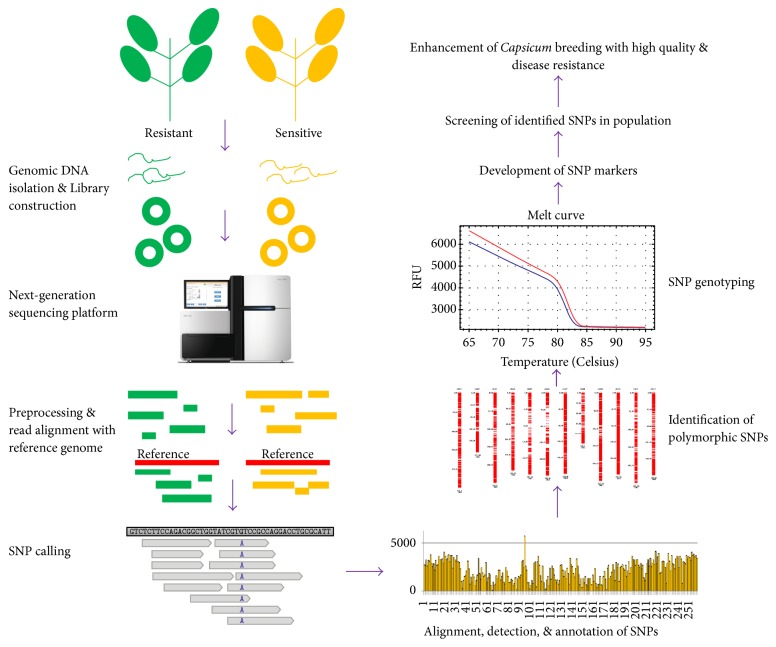
A schematic illustration of application of NGS approach in the genome-wide discovery of SNPs.

The reference genome sequence of* Capsicum annuum* cv. CM334 was sequenced with 186.6X genome coverage by Kim et al. [1] and another sequence of cultivated pepper Zunla-1 has been sequenced by Qin et al. [21] using whole genome shot-gun approach in Illumina platform. The pepper genome is composed of 12 chromosomes with the whole genome size of 3.48 Gb which is larger than the genome size of its close relative tomato [1]. The genome of* Capsicum annum* cv. CM334 incorporated 37,989 numbers of scaffolds with a total length of 3.06 Gb and 34,903 contigs were determined with the overall length of 2.96 Gb [1]. Furthermore, the occurrences of 34,903 genes with the average/total coding sequence length of 1,009.9/35.2 Mb were identified in the genome [1]. In addition, the CM334 genome consisted of 76.4% transposable elements. Among the transposable elements, the majority of them belonged to long terminal repeats (LTR) elements and mostly corresponded to Gypsy elements. Moreover, 177 microRNAs that are categorized into 37 microRNA families were identified in the* Capsicum annuum* cv. CM334 genome. Moreover, the study by Kim et al. [1] has provided deeper insights into the capsaicinoid biosynthesis pathway responsible for the pungency in pepper. According to Qin et al. [21], the reference genome of cultivated pepper* C. annum* Zunla-1 enclosed 967,017 scaffolds. Similar to CM334, the occurrence of 70.3% of LTR elements was observed in the genome of Zunla-1 [21]. Likewise, the numbers of protein coding genes were in similar range (35,336) in comparison to CM334 [21]. The genomic information obtained from these studies aids in the deeper understanding of domestication and evolution of pepper. Moreover, the availability of genomic data facilitates the generation of several useful SNP markers related to vital traits which aid in the improvement of pepper molecular breeding. A schematic illustration of the SNP discovery has been shown in Figure 1 and the detailed impacts of SNPs in pepper have been discussed below.

## 3. Discovery of SNPs Associated with Pungency in* Capsicum*

Next-generation sequencing methodologies permit cost effective and rapid genotyping by sequencing, which facilitates the discovery and validation of SNP markers in several crops. Among the NGS approaches, the transcriptome assembly can be utilized for the generation of high-quality DNA markers that are vital to uncover the genetic variations in pepper [22]. Previously, Ahn et al. [23] have profiled the transcriptome of* Capsicum* and identified molecular markers in four pepper varieties with contrasting pungency and pigment characteristics using 454 pyrosequencing technology. The study has utilized the EST assemblies reported by Kim et al. [24] as the reference for the assembly and analysis of pepper transcriptome and molecular marker discovery. According to Ahn et al. [23] the variably pigmented “Mandarin” and “Blackcluster” cultivars possessed a total of 1657 SNPs with 1292 homozygous SNPs. Similarly, the highly pungent cultivar “Saengryeg 211” and nonpungent “Saengryeg 213” cultivar consisted of 1851 SNPs with majority of the SNPs classified into homozygous (1319) SNPs which can be utilized for the breeding purposes.

Pungency is the primary property of the pepper which is considered as the vital quality. In pepper the pungency is caused by presence of alkaloids called capsaicinoids [25]. In general, the capsaicinoids are synthesized in the placental tissues of pepper by the action of several enzymes. The notable enzymes involved in the capsaicinoids biosynthesis are acyl-CoA synthetase (ACS), acyl carrier protein (ACL), 4-coumarate CoA ligase (4CL), coumaroyl shikimate/quinate 3-hydroxylase (Ca3H), phenylalanine ammonia lyase (PAL), cinnamic acid 4-hydroxylase (Ca4H), caffeic acid O-methyl transferase (COMT), putative aminotransferase (pAMT), branched-chain amino acid transferase (BCAT), *β*-ketoacyl ACP synthase (KAS), acyl-ACP thioesterase (FAT), acyl-transferase 3 (AT3), and capsaicin synthase (CSY) [25]. In recent days, the molecular regulation of capsaicinoids biosynthesis has been studied widely to understand the mechanism behind the pungency modulations in pepper. The application of NGS based molecular marker approaches for the identification of pepper cultivars with varying pungency level aids in the development of new cultivars. In* Capsicum frutescens* L., the RNA-seq analysis of pericarp and placenta predicted three novel genes such as dihydroxyacid dehydratase (DHAD), threonine deaminase (TD), and prephenate aminotransferase (PAT) involved in the capsaicinoids biosynthetic pathway [22]. Moreover the report also identified a total of 9,150 SNP markers in* C. frutescens* and* C. annuum* for the analysis of polymorphism among the pepper varieties with different pungency levels [22]. Among the SNPs, the transition type (A-G) dominated the other types of SNPs. Similarly, a pungency related SNP (G/T) has been detected from the expressed sequence tags (EST) of placenta specific SB2-66, a cDNA clone of AT3 enzyme involved in the capsaicinoid biosynthesis pathway [25]. The AT3 encodes for a vital pungency gene called* Pun1*, the* Capsicum* cultivars with recessive form of this gene* (pun1)* with 2.5 kb deletion results in the nonpungency [25]. Therefore, determination of molecular markers in close proximity to the gene could distinguish the pepper plants with different pungency property. Garcés-Claver et al. [25] have implemented tetra-primer amplification refractory mutation system-PCR and cleaved amplified polymorphic sequence methods to develop the SNP marker. Moreover, the identified SNP marker significantly distinguished 29 cultivars (19 nonpungent and 10 pungent) of* Capsicum annuum*. The nonpungent cultivars consisted of G allele whereas the pungent cultivars possessed T alleles. Furthermore, the determined SNP marker can also be correlated to the phenotypic characteristics of* Capsicum* species such as* C. baccatum, C. cardenasii, C. chinense, C. eximium, C. frutescens, C. galapagoense,* and* C. tovarii *[25].

A recent study by Nimmakayala et al. [26] generated 66,960 SNPs using GBS approach with 1189 haplotypes consisting of 3413 SNPs. Moreover the study has incorporated the principal component analysis and Bayesian model dependent population structure analysis which revealed the importance of capsaicin content and fruit weight characters in determining the pepper genome. In addition, the genome-wide association studies determined the biomarkers linked to capsaicinoid synthesis such as Ankyrin-like protein, IKI3 family protein, ABC transporter G family, and pentatricopeptide repeat protein and 16 SNP markers associated with the genes regulating the fruit weight trait [26]. Similarly, the elucidation of gene specific polymorphism and SNP analysis associated with the capsaicin biosynthesis pathway by Reddy et al. [27] aided in the development of markers related to capsaicinoid metabolic pathway in pepper. The report suggested that the gene* Pun1* acts as the vital regulator in the capsaicinoid pathway which influences the production of capsaicinoids and its precursors [27]. In addition, the study has identified six SNPs in the promoter sequence of* Pun1 *gene linked with capsaicin synthesis in plants [27].

Taken together, the discovery of SNP markers in pepper associated with the pungency trait could render novel information on the genes involved in the biosynthesis of capsaicinoids.

## 4. Discovery of SNPs Associated with Bacterial Wilt Resistance in* Capsicum*

The pepper production has been drastically hindered by diseases, for instance, the bacterial wilt (BW), caused by* Ralstonia solanacearum*, is one among the most damaging plant diseases [28]. In particular, it leads to heavy yield loss in Solanaceae plants, including pepper. Although various physical and chemical agents have been applied extensively to prevent bacterial wilt, the incorporation of genetic resistance to the susceptible cultivars by means of novel genomics-assisted breeding approaches could be considered as the promising tool to circumvent the problem of bacterial wilt in pepper. The molecular breeding technique to impart bacterial wilt resistance is the valuable, feasible, and eco-friendly solution [29]. Determination of SNP markers associated with the bacterial wilt resistance will aid in the development of novel pepper varieties with enhanced resistance to bacterial wilt disease. Moreover, the marker assisted selection in molecular breeding of crops facilitates the pyramiding of several genes into a single cultivar [30]. Further, the availability of the complete genome sequence information of pepper improves the discovery of molecular markers linked to bacterial wilt resistance. Recently, a genome-wide discovery of SNPs has been accomplished by Ahn et al. [31] using whole genome resequencing approach. The genome of two pepper varieties, namely, Saengryeg 211 (susceptible) and 82PR66 (resistant), with different bacterial wilt resistant property has been resequenced and compared with the* C. annuum* cv. CM334 reference sequence. The whole genome resequencing resulted in 118,588,231 and 148,774,861 paired raw reads for Saengryeg 211 and 82PR66. In addition, the susceptible variety consisted of 6,804,889 SNPs whereas the resistant variety contained 4,293,534 SNPs [31]. Furthermore, the study resulted in the identification of 5,514,563 polymorphic SNPs between Saengryeg 211 and 82PR66. The discovered SNPs were classified into homozygous, heterozygous, and other types in both the varieties. Among the SNPs discovered in Saengryeg 211, 95.04% have been classified as homozygous and 3.05% as other types followed by 1.91% heterozygous SNPs. Similar pattern of SNP types was also observed in the 82PR66 variety [31]. According to Ahn et al. [31], the distribution of SNPs varied between the chromosomes with the highest density of SNPs occurring in chromosomes 10 and 11 for Saengryeg 211 and 82PR66, respectively. On the other hand the least number of SNPs was identified in the chromosome 8 in both the pepper varieties [31]. In addition, the discovered SNPs have been categorized based on its distribution in the pepper genome as intergenic, genic, and intronic SNPs. The majority of the SNPs were identified in the intergenic region in both Saengryeg 211 (93.39%) and 82PR66 (98.10%) followed by genic region. Apart from the above-mentioned SNPs, Ahn et al. [31] determined the 2039 SNPs associated with the nucleotide binding site leucine rich repeat (NBS-LRR) loci. In general, most of the disease resistance genes belong to the NBS-LRR family. Overall, the genome-wide discovery of SNPs related to bacterial wilt resistance could enhance the molecular breeding of pepper with bacterial wilt resistance.

## 5. Discovery of SNPs Associated with Blight Resistance in* Capsicum*

Transcriptomics present an opportunity to discover crucial genes associated with a particular resistance trait based on the identification of numerous DNA based markers or by the discovery of differentially expressed genes [32]. Globally, in pepper breeding, identification of disease resistance is emerging as the quality of concern.* Phytophthora capsici* is a destructive fungal pathogen that can affect* Capsicum* in all developmental stages and tissue types and causes blight disease [33, 34]. The molecular rationale behind the blight resistance mechanism has been still under elucidation; however, a mere amount of information exists on the quantitative trait loci (QTLs) related to the resistance mechanism [35]. A study by Lu et al. [35] has attempted to identify the molecular markers linked to the* P. capsici* resistance candidate genes in pepper using Roche 454 pyrosequencing technology in two pepper varieties of* C. annuum*, YCM334 (resistant variety) and Taean (susceptible variety). From the two pepper varieties with contrasting* P. capsici* resistance property, a total of 11,133 SNPs with 3281 high-confidence polymorphic SNPs were discovered [35]. Thus, the SNPs discovered can be utilized for the subsequent identification of* P. capsici* resistance-related genes via map-based cloning strategies.

## 6. Discovery of SNPs Associated with Cucumber Mosaic Virus (CMV) Resistance in* Capsicum*

Cucumber mosaic virus (CMV) is a pathogen with broad range of host and is widely transmitted by aphid infection [36]. The CMV virus causes heavy loss in the Solanaceae crops especially in pepper. However, in* Capsicum*, many resistance sources have been discovered among various species but majority of the resistant varieties exhibit partial resistant trait directed by multiple genes [37–41]. The mechanism of CMV resistances ranges from suppression of viral replication to cell to cell inhibition [37, 42, 43]. For the first time, three SNP markers linked to CMV resistance genes such as* Tm-1* (tomato mosaic resistant-1) and* Cmr1* (cucumber mosaic resistance 1) have been identified by Kang et al. [44] in* C. annuum* “Bukang.” The* Tm-1* gene belongs to tomato identified in the chromosome 2 which renders broad range of resistance against the viruses particularly tomato mosaic virus [44]. Similarly, the* Cmr1* gene is a single dominant gene discovered in pepper that induces resistance against CMV disease [44]. According to Kang et al. [44], among the identified SNP markers, CaTm-int3HRM is situated in close proximity (2cM) to the* Cmr1* gene which distinguished the presence of polymorphism among the F_2_ population of* Capsicum*. The SNP marker discovered for the CMV resistance can be highly useful for the discrimination between CMV sensitive and resistant pepper cultivars [44]. In addition, the SNP marker information can be extrapolated for the discovery of novel molecular markers which lie close to the resistant genes.

## 7. Discovery of SNPs Associated with Anthracnose Resistance in* Capsicum*

Anthracnose is a predominant fungal disease caused by* Colletotrichum* species in pepper plants grown worldwide; however the highest rate of infection has been reported in Asia [45]. In rainy season, the occurrence of anthracnose is inevitable which leads to pre- and postharvested fruit yield losses [46]. In order to overcome the problem of anthracnose, molecular marker assisted breeding of pepper has been considered as the vital approach. Recently, SNP markers related to anthracnose resistance QTLs have been generated in two* Capsicum* populations such as* Capsicum annuum* “Bangchang” ×* C. chinense* “PBC932” and* C. baccatum* “PBC80” × “CA1316” by Mahasuk et al. [47]. In* C*.* annuum* “Bangchang” ×* C. chinense* “PBC932” the high throughput detection of SNPs using Kompetitive Allele Specific PCR method resulted in the development of 1024 SNPs of which 288 displayed significant polymorphism among the parents [47]. Similarly, a total number of 1165 SNPs were generated from the* C. baccatum* “PBC80” × “CA1316” which resulted in 510 polymorphic SNPs [47]. Further, the identified SNPs were utilized for the analysis of anthracnose resistance QTL in both* C. annuum* species. The SNPs derived from the* C*.* annuum* “Bangchang” ×* C. chinense* “PBC932” aided in the identification of two anthracnose resistance QTLs (RA932 g and RA932r), whereas three major QTLs responsible for anthracnose resistance (RA80rP2, RA80rP3.1, and RA80rHP1) have been located with the SNP marker information in* C. baccatum* “PBC80” × “CA1316” [47]. Overall, the SNP markers linked to the anthracnose resistance will aid in the screening of anthracnose disease resistance and sensitivity in the pepper which greatly benefits the molecular breeding of pepper with disease resistance.

## 8. Conclusions

Next-generation sequencing (NGS) techniques have revolutionized the field of plant genomics. The advent of NGS has resulted in high throughput sequencing of numerous agricultural and horticultural plants with economic importance. Moreover, the availability of robust genomic and bioinformatics methods enhanced the understanding of trait improvements in various important crops. In the present review, a comprehensive overview on the discovery of single nucleotide polymorphism (SNP) markers associated with different traits such as pungency, bacterial wilt resistance, blight resistance, cucumber mosaic virus resistance, and anthracnose resistance of pepper have been demonstrated. However, in the future, the developments in the field of plant genomics will pave the way for the implementation of vital tools which will assist in the breeding of pepper.
